# Characterization of a L136P mutation in Formin-like 2 (FMNL2) from a patient with chronic inflammatory bowel disease

**DOI:** 10.1371/journal.pone.0252428

**Published:** 2021-05-27

**Authors:** Raphael Trefzer, Orly Elpeleg, Tatyana Gabrusskaya, Polina Stepensky, Hagar Mor-Shaked, Robert Grosse, Dominique T. Brandt

**Affiliations:** 1 Institute of Pharmacology, University of Marburg, Marburg, Germany; 2 Department of Genetics, Hadassah-Hebrew University Medical Center, Jerusalem, Israel; 3 Department of Gastroenterology, St. Petersburg State Pediatric Medical University, St. Petersburg, Russia; 4 Department of Bone Marrow Transplantation and Cancer Immunotherapy, Hadassah-Hebrew University Medical Center, Jerusalem, Israel; 5 Department of Genetics, Hadassah-Hebrew University Medical Center, Jerusalem, Israel; 6 Institute of Experimental and Clinical Pharmacology and Toxicology, University of Freiburg, Freiburg, Germany; University of Illinois at Chicago, UNITED STATES

## Abstract

Diaphanous related formins are highly conserved proteins regulated by Rho-GTPases that act as actin nucleation and assembly factors. Here we report the functional characterization of a non-inherited heterozygous FMNL2 p.L136P mutation carried by a patient who presented with severe very early onset inflammatory bowel disease (IBD). We found that the FMNL2 L136P protein displayed subcellular mislocalization and deregulated protein autoinhibition indicating gain-of-function mechanism. Expression of FMNL2 L136P impaired cell spreading as well as filopodia formation. THP-1 macrophages expressing FMNL2 L136P revealed dysregulated podosome formation and a defect in matrix degradation. Our data indicate that the L136P mutation affects cellular actin dynamics in fibroblasts and immune cells such as macrophages.

## Introduction

Over the last decade many research efforts were devoted towards identifying genetic components of intestinal inflammation. Genome-wide association studies (GWAS) addressed the identification of IBD-related genes and found over 200 gene loci that were associated with developing Crohn’s disease (CD) or Ulcerative colitis (UC) [1–5]. These findings pictured the wide field of genetic influence on IBD that has to be further evaluated regarding the relevance in individual cases. The complex pathophysiology of CD is not completely understood. However, recent reviews could outline genetic influence as well as various environmental and lifestyle factors that contribute to the manifestation of a chronic bowel disease [6]. In the last few years several studies on IBD genetics pointed towards the relevance of immunoregulation defects in the pathomechanisms of IBD [4,7,8]. Especially in early-onset pediatric CD immunodeficiencies seem to play a central role [8].

FMNL2 is a member of the family of diaphanous-related formins (DRF) which control actin nucleation and assembly and are characterized by a conserved formin homology domain (FH2) [9,10]. For most formins it has been shown that under basal conditions the protein is held in an autoinhibited configuration via an intramolecular interaction between the C-terminally located DAD (Dia autoinhibitory domain) and the DID (Dia inhibitory domain) at the N-terminus, which can be released through e. g. binding of Rho-GTPases [11]. FMNL2 is expressed in a wide number of human tissues such as the nervous system, the gastro-intestinal tract or the lymphatic system [12]. Several *in vitro* studies showed the relevance for FMNL2 in a variety of cell functions that require reorganization of the actin cytoskeleton such as the initiation of epithelial cell-cell-contacts [13], the formation of Filopodia [14] or the turnover of β-Integrins for invasive cell migration [15]. In a clinical context, upregulation of FMNL2 is reported to drive tumor progression and increase metastatic behavior in colorectal cancer (CRC) and melanoma [16,17]. Formin mutations can result in various forms of pathologies like the Charcot-Marie-Tooth-Syndrome and Focal Segmental Glomerulosclerosis (FSGS) which are associated with mutations of INF2 [18,19]. Despite their relevance in various actin-dependent cell functions, a role of formins in the pathomechanisms of IBD and immune defects has not yet been described.

Here, we report the first characterization of a formin mutation that is associated with inflammatory bowel disease (IBD).

## Material & methods

### Exome sequencing analysis

Trio exome analysis was performed on DNA of the patient and his parents. Exonic sequences were enriched with the SureSelect Human All Exon 50 Mb V5 Kit (Agilent Technologies). Sequences were generated on a HiSeq2500 (Illumina) as 125-bp paired-end runs. Read alignment and variant calling were performed with *DNAnexus* using default parameters with the human genome assembly hg19 (GRCh37) as reference.

Exome analysis of the patient yielded 50.6 million mapped reads with a mean coverage of 88×. Following alignment to the reference genome (hg19) and variant calling, variants were filtered out if the total read depth was less than 8 × and if they were off-target (>8 bp from splice junction), were synonymous, or had minor allele frequency (MAF) > 0.5% in the *genomAD* database (Genome Aggregation Database, Cambridge, MA).

### Material, cloning and antibodies

Cell culture reagents were purchased from Invitrogen. All other reagents were from Sigma- Aldrich if not stated otherwise. Restriction enzymes and PCR reagents were purchased from Thermo Scientific.

FMNL2 derivates were based on human FMNL2. Side directed mutagenesis was performed by the overlap extension method [20] and cloned as Xho1/ACC65I fragment into EGFP_N1. Full length FMNL2 GFP variants were isolated by PCR amplification using the following Primers: fwd 5’*GTCGAC*ATGGGCAACGCAGGGAGCATGGATTCGCAG and rvs 5’*CTCGAG*CTACTTGTACAGCTCGTCCATGCCGAGAG and ligated in Entr1A entry vector using Sal1 and Xho1 restriction enzymes. FMNL2 cDNAs were then inserted into the lentiviral expression vector pIND20 [21] by recombination using the Gateway LR Clonase (Invitrogen). For cloning in pWPXL expression vector the following primers were used: fwd 5’*ACGCGT*ATGGGCAACGCAGGGAGCATGGATTCGCAG and rvs 5’*ACTAGT*CTACTTGTACAGCTCGTCCATGCCGAGAG and the PCR fragment was ligated via Mlu1 and Spe1 restriction sites. For cloning of a flag-tagged N-terminal FMNL2 (aa 23–484), the following primers were used: fwd 5’*CTCGAG*ATGGGCAACGCAGGGAGCATGGATTCGCAG and rvs 5’ *GCGGCCGC*TCACTTGTCGTCATCGTCTTTGTAGTCGCCGTCT. The PCR fragment was ligated via Xho1 and Not1 in the N1_EGFP (Clontech) vector backbone. The FMNL2 C-terminal fragment (aa 521–1092) has been described [15].

Anti-Flag HRP (A8592), anti-Myc HRP (16–213) and the anti-Flag M2 Affinity Gel (A2220) were obtained from Sigma-Aldrich. The mouse anti-GFP HRP antibody (AB_247003) was obtained from Miltenyi Biotec, the mouse anti-Vinculin (MAB3574) was from Millipore and the goat anti-mouse Alexa-Fluor® 647 antibody (ab150115) was from Abcam.

### Cell culture and lentiviral transduction

HEK-293T cells, NIH/3T3 cells and HeLa cells were cultured in DMEM containing 10% fetal bovine serum at 37°C at a 5% CO_2_ atmosphere. THP-1 cells were maintained in a suspension culture in RPMI supplemented with 10% fetal bovine serum at a concentration of 200.000–600.000 cells/ml. Differentiation of THP-1 cells into a macrophage state was achieved by a treatment with PMA 25nM for 24–48 hours [22].

For the generation of lentiviral particles, HEK-293T were transfected with the pInducer or pWPXL plasmids together with the packaging plasmids psPAX and pMDG.2 using calcium-phosphate precipitation. After 72 hours, supernatants containing viral particles were harvested and filtered through 0,45 μm pore filters. Lentiviral particles were concentrated by centrifugation with 10.000 g at 4°C for 4 hours with a sucrose gradient as described previously [23]. NIH/3T3 cells were infected with the concentrated lentivirus for 24 hours. Positive transduced cells were selected by Puromycin treatment at 3μg/ml for 5 days. For the induction of protein expression, Doxycyclin was used at 1ng/ml for 24 hours. For stable co-expression of LifeAct mCherry, pInducer-transduced selected cells were transduced with pWPXL LifeAct mCherry in the same matter.

Stable THP-1 cell lines expressing pWPXL FMNL2 GFP variants were generated by spininocculation. Infection was successful by centrifuging the THP-1 cells with the concentrated lentivirus for 2 hours with 2500 g at 37°C. To ensure a homogenous expression amongst the cell population cells were sorted using FACS.

### Co-immunoprecipitation and Western blotting

Standard Western blotting and Co-Immunoprecipitation was performed as described previously [15]. HEK-293T cells were transfected with the constructs of interest using calcium-phosphate. After 24 hours, cells were lysed in ice cold Triton based buffer (Tris 20 mM, NaCl 150 mM, EDTA 2 mM, Triton-X-100 0,1%) supplemented with complete protease inhibitors (Roche). Proteins were isolated by centrifugation at 13.000 g for 10 minutes at 4°C. 90 μl of the lysates were diluted in 30 μl 4X SDS-containing sample buffer for expression control via immunoblot in the further course. For immunoprecipitation lysates were incubated with Flag-M2 Agarose Affinity Beads (Sigma) and mixed by rotation for 1 hour at 4°C. Beads were washed three times by spin-down sedimentation at 4°C, gentle aspiration of the supernatant and dilution in 1 ml fresh ice-cold lysis buffer. Complexes were eluted from the beads with SDS-containing sample buffer at 95°C for 5 min. Eluted proteins were then resolved by SDS-PAGE and subjected to nitrocellulose membranes for immunoblot analysis using horseradish peroxidase-conjugated anti-Flag and anti-Myc antibodies (Sigma) at a 1:5000 dilution. HRP signal was detected on Medical X-Ray films (Fuji) using X-Ray Film Processor (Medical Index).

### SRF luciferase assay

NIH/3T3 cells at a low passage were seeded on 6-well plates and transfected with 0,5 and 1 μg of the respective FMNL2 constructs together with 0,1 μg pDa.Fos and 0,2 μg pRL-TK using Lipofectamine 2000 (Invitrogen) reagent. After 24 hours cells were starved in 0,5% fetal bovine serum for 24 hours. On the day of the experiment cells were lysed in ice cold Triton based buffer for 10 minutes, scraped and centrifuged at 13.0000 rpm for 15 minutes 4°C. Firefly and Renilla luciferase activity were measured successively using a Luminoscan Ascent Microplate Luminometer (Thermo Scientific) with the Ascent Software (Thermo Scientific) at an integration time of 800 ms. Firefly Luciferase activity values were normalized to Renilla activity.

### Fluorescence microscopy and live cell imaging

For fluorescence microscopy analysis cells were kept on glass cover slips. Cells were fixed with 8% PFA and stained with Phalloidin Alexa 555 (1:200) or 647 and DAPI (0,3 μg/ml). For antibody staining the cells were permeabilized with 0,3% TX for 5 min, blocked with 5% FBS and incubated with the indicated primary antibodies at 4°C overnight. Secondary antibodies were incubated for 1 hour at room temperature. Images were taken using a confocal laser microscope (LSM 700, Carl Zeiss). All single cell images and image sequences were taken with a 63x/1.4 objective (Carl Zeiss) using immersion oil. Tile scans were taken using a 40x objective (Carl Zeiss) choosing 9 picture frames per scan. Imaging was done in Z-stacks, the layers were adjusted to the appropriate experiment and maximum intensity projections were created using the ZEN Software.

For live cell imaging cells were kept in an incubation chamber at 37°C and 5% CO_2_. Sequential images were taken at the frequency indicated in the appropriate figure legends in Z-stacks of 0,5 μm. Maximum intensity projections were created afterwards in the ZEN Software. Further analysis was done in FIJI.

To quantify filopodia, HeLa cells were kept on acid-treated glass coverslips and transfected with the GFP-tagged formin variants. After fixation, staining and imaging as described above, filopodial structures of at least 10 cells per condition were analyzed in FIJI using the *multi point tool*. For quantification and length measurements of filopodia, Phalloidin 555 nm channels were adjusted to maximum brightness/contrast levels to ensure equivalent sensitivity of filopodia detection.

For the imaged based quantification of the number of podosomal structures per cell, THP-1 cells were kept on untreated glass coverslips and were treated with PMA for 48 hours as stated above. Cells were fixed and stained with phalloidin and a vinculin antibody. In 4 independent experiments 2 random tile scans were taken for each condition. Subsequently, vinculin-positive ring structures of at least 58 cells per condition were quantified using the *multi pointing tool* in FIJI.

### Live cell spreading analysis

For Live Cell Imaging of fibroblasts, stably transduced NIH/3T3 cells were trypsinized and transferred to untreated 8-well μ-slides (Ibidi) at a density of 1,5 x 10^4^ cells/cm^2^. Cells were sedimented to the bottom and imaging was started when cells started adhering. To ensure visualization of the spreading process to a full extend, 10 Z-stacks were set with the lowest two stacks being underneath the bottom of the dish. Over a course of 120 minutes Z-stack images were taken every 6 minutes using a 63x/1.4 objective with immersion oil to record the dynamics of the spreading process. For detection of GFP signal a 488 nm channel was used, LifeAct was imaged in 555 nm. The created Maximum Intensity Projections were adjusted in FIJI and filopodial structures were quantified as stated above in the stills of the indicated time points.

### Impedance measurements of cell spreading

5.000 stably transduced NIH/3T3 cells were seeded into 96 well E-plates in triplicates, respectively. The cells were left to sediment and adhere for about 30 minutes, after that the base-time was set. After initial normalization of the impedance values, automated sequential impedance measurements were done every 15 minutes. The spreading of the cell bodies results in a reduction of electron flow between electrodes on the bottom of the wells. This could be detected using a xCELLigence Single Plate System (ACEA Biosciences) which was kindly provided by the lab of Moritz Bünemann (BPC University of Marburg). The measured relative cell impedance was calculated by the appropriate software into a normalized cell index, which indicates the relative increase of cell impedance from the set base-time. The normalized cell index was plotted to picture the dynamic rise of impedance which correlated to the increase of the mean cell area.

### Image-based analysis of spreading area

NIH/3T3 cells were trypsinized and differentiated THP-1 cells were diluted by gentle scraping. 4 x 10^4^ cells were subsequently transferred onto untreated 12 mm glass coverslips and were incubated to adhere and spread for the indicated time periods. After fixation and staining with Phalloidin 555 as stated above, 2 random tile scans were taken for each condition with a sample size of more than 50 cells per experiment. The cell area was measured in the 555 nm channel as the Phalloidin mediated actin staining allowed clear definition of the cell edges across all conditions. Cell areas of all the imaged cells were determined using the *freehand selections tool* in FIJI. Cells that were cut off at the image edges were excluded from the quantification.

### Matrix degradation assay

12 mm glass cover slips were coated with Oregon 488 conjugated gelatin (Invitrogen) as described previously [24]. Briefly, coverslips were incubated with 50 μg/ml Poly-L-Lysine (Sigma) for 15 minutes and fixed with 0,5% Glutaraldehyde (Sigma) for 10 minutes at room temperature. After repeated washing with PBS, the cover slips were quenched with Sodium Borohydride (Sigma) at 5mg/ml for 10 minutes and washed again. Oregon 488 conjugated Gelatin was diluted to a working solution of 0,2 mg/ml in PBS. After heating the gelatin solution to 50°C the coverslips were inverted on a 50 μl drop of gelatin and left to polymerize for 15 minutes at room temperature, protected from light.

For the experiment, 150.000 THP-1 cells expressing the indicated constructs were seeded on gelatin-coated coverslips in the well of a 12-well plate. The assay medium contained 25 nM PMA to differentiate THP-1 cells into a macrophage state [22]. After 24 hours the cells were fixed with 8% PFA for 10 min, permeabilized with 0,3% TX for 5 min and stained with Phalloidin 555 (1:200) and DAPI (0,3 μg/ml). Cells were imaged with a confocal laser microscope (LSM 700, Carl Zeiss) and quantified with ImageJ. In each of the 3 independent experiments 2 Random tile scans were taken for each condition. In total a sample size of at least 100 cells per experiment were analyzed, respectively. Black areas in the green fluorescent matrix were counted as degraded areas. The fraction of cells featuring a degraded area were quantified and defined as “cells degrading matrix”.

### Image processing and statistical analysis

Maximum intensity projections from Z-Stacks and creating subsets of parallel imaged movies were done using ZEN Software (Zeiss, Jena). Adjustments of brightness and contrast on confocal images and the adding of scale bars was done in FIJI. Further quantification analysis of image data was performed as described in the respective sections.

Statistical analysis was performed using Prism GraphPad. Statistical significance was calculated using an unpaired students t-test to compare two groups.

### Ethics statement

Ethical approval was not sought for the present study because all data were collected from *in vitro* experiments which were only performed with immortalized cell lines. The report about the clinical course is kept in an anonymous form. Written informed consent was obtained from the mother of the mutation carrier pursuant to the Consent Form for Publication in a PLOS Journal.

## Results and discussion

FMNL2 p.L136P was newly identified in a clinical case of pediatric Crohn’s disease (CD). The male patient presented with significant intestinal manifestations from the first year of his life. At the age of 6 months, he showed signs of failure to thrive (FTT), abdominal distention and bloody diarrhea. Throughout his childhood, he suffered from a severe clinical course with FTT, abdominal pain and intermittent bloody diarrhea. Endoscopic evaluation revealed hemorrhagic colitis, perianal abscess and severe inflammation of the upper gastrointestinal tract. Furthermore, at the age of seven years he developed chronic nonbacterial osteomyelitis of the right tibia which required surgical treatment with resection of the distal metaphysis and alloplastic reconstruction. Considering the number of infectious complications such as severe bronchopneumonia at the age of 3 and persisting EBV-infection, the treating physicians suggested an underlying primary immunodeficiency. Routine immunological workup including immunoglobulin levels and immunophenotyping was normal. Through immunosuppressive therapy with prednisone and azathioprine the inflammation was partially controlled, yet the patient showed still signs of IBD. In the further course of the disease, treatment with infliximab, methotrexate and adalimumab caused a significant improvement in clinical, laboratory and endoscopic findings. Now, the child is nine year old boy with well controlled CD but still suffering from moderate FTT. As a part of diagnostic workup whole exome sequencing (WES) was done. A heterozygous FMNL2 p.Leu136Pro mutation was identified by WES of a peripheral blood sample and was found to occur de novo, i.e. was not inherited from the parents. It is therefore predicted to be “likely-pathogenic” (https://varsome.com/variant/hg19/FMNL2%20Leu136Pro). Whether this mutation would result in gain- or loss-of-function (LOF) was unclear at this stage; the gene pLI (probability of being loss-of-function intolerant), calculated by comparing the observed number of LOF among gnomAD individuals to the expected number based on the protein length, was rather high (expected 55, observed 9, pLI-0.99), suggesting that the protein is intolerant to haploinsufficiency (heterozygous LOF mutations). The data presented herein suggest that this variant is responsible for the patient phenotype by a gain-of-function mechanism.

### Sequencing analysis

Following alignment of exonic reads and variant filtering as described under Methods, 185 variants remained. Among them there were no compound heterozygous, hemizygous, or homozygous variants in relevant genes. However, when compared to the parents variant lists, a single heterozygous non-inherited exonic variant was noted. This was *Hg19 Chr*.*2*: *153415301T>C*, *c*.*407T>C*, p.Leu136Pro in the *FMNL2* gene. This variant was not carried by any of the ~124,000 individuals whose exome analysis was deposited in gnomAD, nor among the 6,000 individuals that constitute the in-house database (Hadassome).

### FMNL2 p.L136P is a deregulated protein and disturbs actin-dependent cell functions

**Fig 1:**
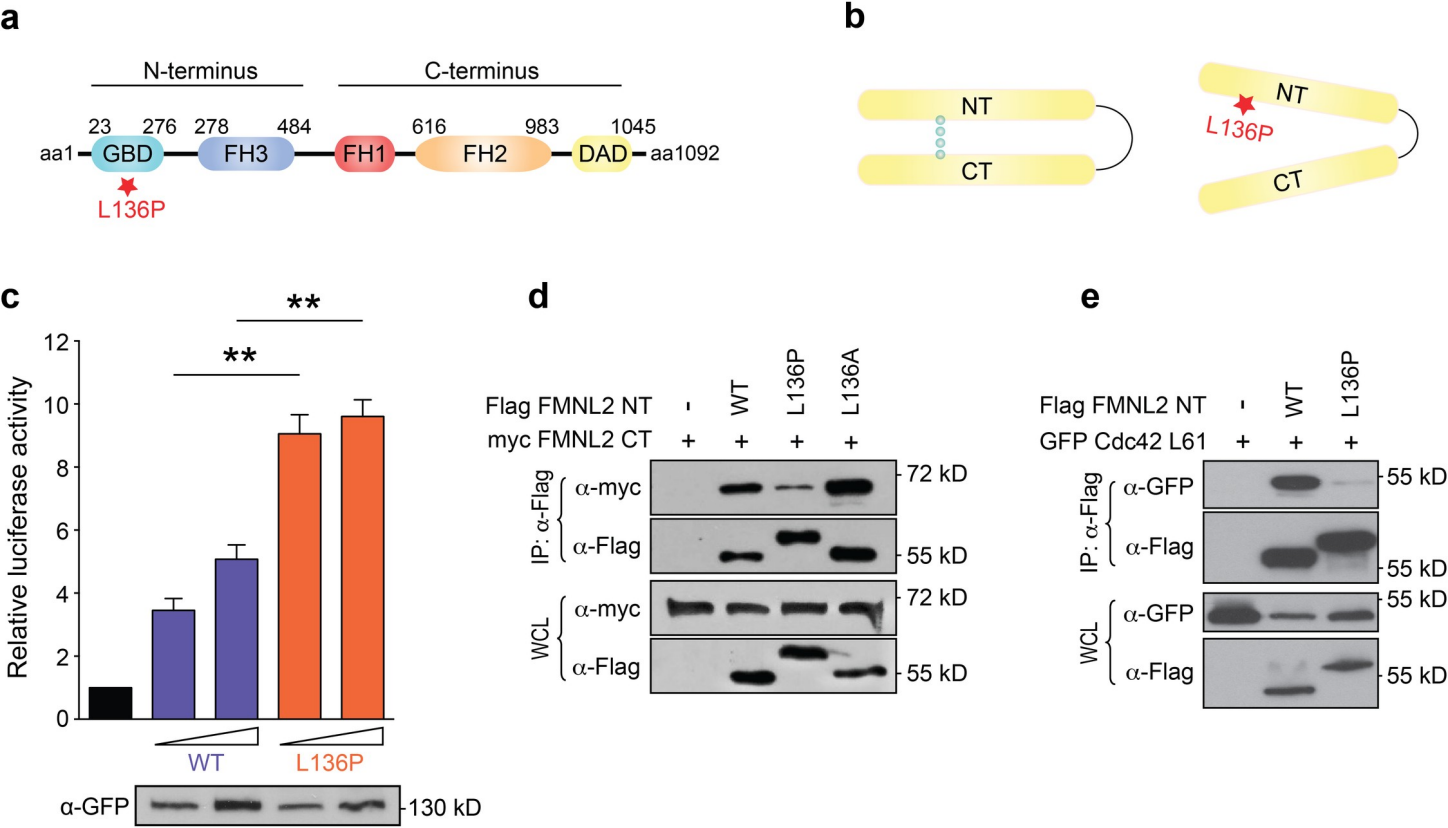
FMNL2 L136P protein regulation is dysfunctional. **a)** Schematic structure of FMNL2. *GBD = GTPase binding domain*, *FH = formin homology domain*, *DAD = diaphanous inhibitory domain*. The asterisk indicates the position of the mutation. **b)** Autoinhibition of Formins is mediated by the binding of N-terminus and C-terminus through interaction of the DID and DAD region. The p.L136P variant disturbs the autoinhibitory mechanism. **c)** SRF Reportergen Assays were performed to assess the protein activity of the FMNL2 GFP variants. NIH/3T3 cells were transfected with pDa.Fos 0,5 and 1,0 μg of the indicated FMNL2 variants. Reportergen activity correlates with formin-mediated SRF activation. FMNL2 L136P shows increase in basal activity (WT_low_ vs. L136P_low_: *p = 0*,*0014*; WT_high_ vs. L136P_high_: *p = 0*,*0029*). Luciferase activity is shown as fold over control sample (black bar, empty vector control) and mean + SEM of 3 independent experiments. Students T-test was used to analyze the statistical difference between two groups (* = *p<0*,*05*; ** = *p<0*,*01*). The immunoblot shows the different expression levels of the indicated GFP-tagged formin variants in the samples used for SRF Assay. **d)** Co-Immunoprecipitation of FMNL2-NT and -CT. HEK-293T cells were transfected with myc-tagged FMNL2 CT and the indicated flag-tagged FMNL2 NT variants. Binding of the two protein fragments was assessed through immunoprecipitation of the flag-NT. Whole cell lysates (WCL) served as expression control as shown in the two lower panels. The interaction of the p.L136P N-terminal fragment with FMNL2 CT is reduced. **e)** Co-Immunoprecipitation of FMNL2 NT and GFP-tagged Cdc42. Immunoprecipitation of the flag-NT was performed on lysates of HEK-T293 cells expressing constitutive active Cdc42 and the indicated FMNL2 NT variants. L136P NT shows a distinct diminished Cdc42 binding.

The position of p.L136 in the FMNL2 protein is localized within FH3 domain which is composed of a dense meshwork of alpha helices, the so-called armadillo repeats [14]. The FH3 domain harbors critical sequences for the regulation of the formin protein, such as the DID domain, which is required for the interaction with the C-terminally located DAD domain and keeps the protein in an inactive conformation (Fig 1A). But also, upstream regulators such as CDC42 require an intact FH3 domain for binding to FMNL2 [14].

Proline is characterized as an amino acid with unusual structural features which are caused by the R-group, that folds back on itself to form a ring with the amino group. That feature can change the bonding angle of a polypeptide chain and can cause a kink in alpha-helices. Hydrogen bonds (H-bonds) between amino- and carboxy-groups at the backbone of an alpha-helix are essential for the structural integrity of the alpha-helix. The kink that can be caused by the Proline R-group disturbs H-bonding and can lead to the structural destabilization of the alpha-helix. As such prolines are often defined as “helix-breakers” [25–27]. We therefore speculated that the mutant formin protein might display altered regulation properties, such as an impaired autoregulation (Fig 1B).

To test for changes in regulation of the mutated FMNL2 we assessed the basal activity towards actin using the SRF-reporter gene assay, which is suited for the quantitative assessment of changes in actin dynamics [28]. Our data reveal that the basal activity of the mutant protein was significantly higher than the wt configuration (Fig 1C), indicating a disturbed autoinhibition. To test this hypothesis, we investigated whether the intramolecular interaction between the N- and C-terminus is affected in the mutant protein. Co-immunoprecipitation of C- and N-terminal fragments of FMNL2 revealed that binding of the C-terminus is drastically reduced when L136 is replaced by proline (Fig 1D). Interestingly the change of the proline seemed to be responsible for this effect, since a N-terminal fragment with an alanine mutation at position L136 did not show a decrease in binding affinity for the C-terminal fragment. Structural changes due to the proline substitution are also reflected by the size shift of the L136P N-terminal fragment whereas the alanine mutant migrates normal as the wt (Fig 1D).

The immunoblots are shown as representatives out of 3 independent experiments.

The localization pattern of FMNL2 is controlled by an N-terminal myristoylation at position G2 [29] and Rho-GTPases [30]. Previous studies described Rac1 as an upstream-regulator for recruiting FMNL2 to the sites of newly formed cell-cell-contacts [13], however Cdc42 seems to control FMNL2 plasma membrane localization [30]. As p.L136 lies within the FH3 domain, it seemed unlikely that the mutation might affect N-myristoylation. However, defective binding to Rho-GTPases could be a possible cause for the disturbed subcellular localization of the formin mutant.

Cdc42 is a typical upstream regulator of FMNL2 and requires in addition to the GBD-domain, an intact FH3 domain for binding to FMNL2 [14]. We therefore tested whether also the binding to Cdc42 is affected by the proline substitution at position aa136. Co-immunoprecipitation studies revealed that binding of Cdc42 as a typical upstream regulator is drastically diminished (Fig 1E).

**Fig 2:**
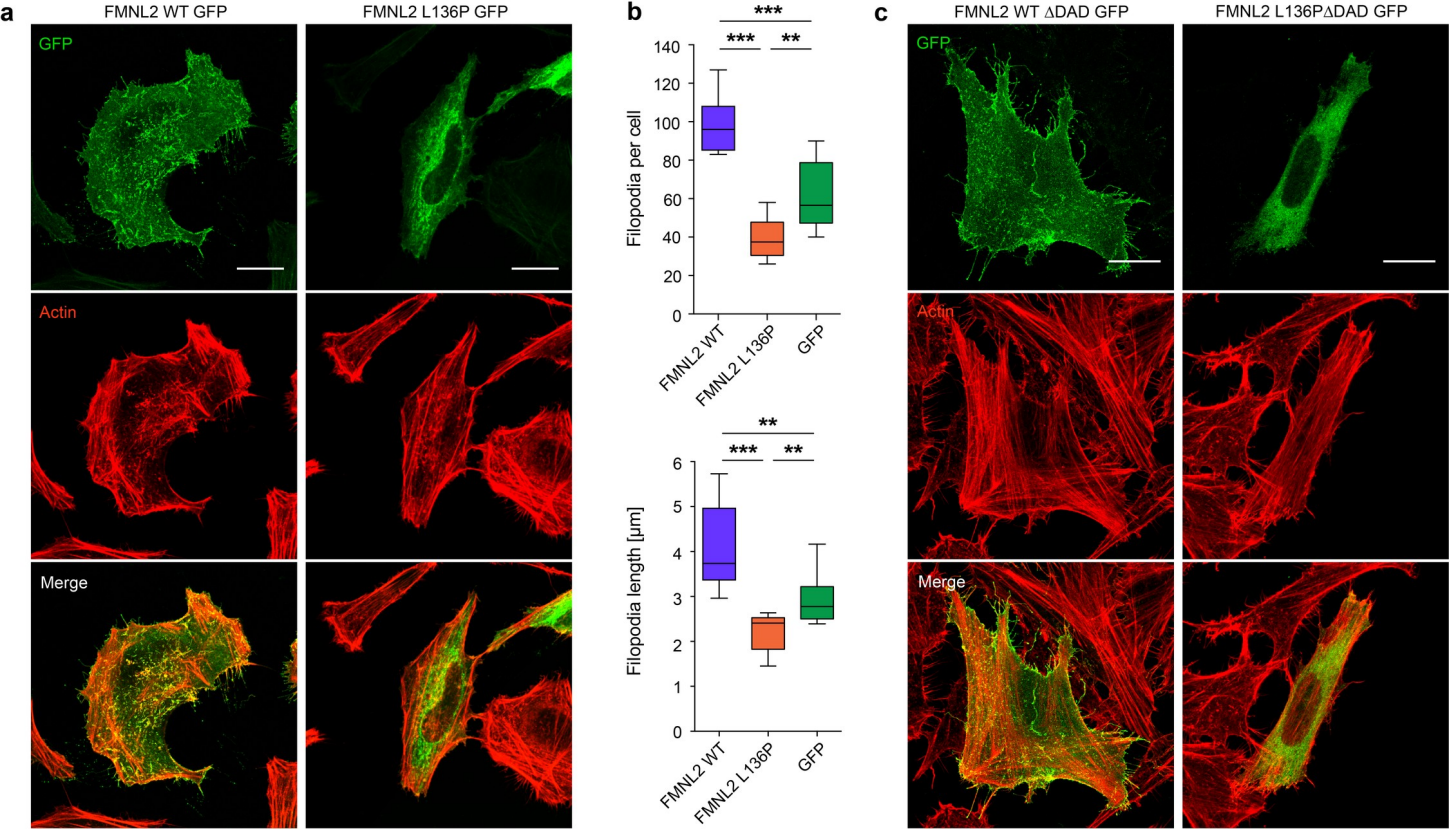
FMNL2 L136P shows an altered localization pattern. **a)** Subcellular localization of the indicated FMNL2 constructs. HeLa cells were kept on acid-treated glass coverslips and transfected with the indicated GFP-tagged FMNL2 variants. Following fixation, cells were stained with phalloidin to visualize F-actin. Scale bar = 20 μm. **b)** Image-based quantification of filopodial structures in the imaged HeLa cells (as shown in a). The transfected WT cells presented with a homogenous filopodia-rich phenotype, whereas the mutant cells all showed obvious loss of filopodia under microscopy. In > 10 cells per condition filopodia were quantified for statistical demonstration. FMNL2 L136P expressing cells show a decrease in number (L136P vs. WT: *p < 0*,*001*; L136P vs. GFP: *p = 0*,*0037*; WT vs. GFP: *p < 0*,*001*) and length (L136P vs. WT: *p < 0*,*001*; L136P vs. GFP: *p = 0*,*0043*; WT vs. GFP: *p = 0*,*001*) of filopodia. Data are shown as median with interquartile range (box) and maximum/minimum (whiskers). Students T-test was used to analyze the statistical difference between two groups (* = *p<0*,*05*; ** = *p<0*,*01*; *** = *p<0*,*001*). **c)** Transient expression of the respective ΔDAD variants in HeLa cells showed no change in the subcellular localization pattern. HeLa cells were kept on glass coverslips and transfected with the indicated constructs. Depletion of the DAD results in a disinhibited formin.

We next analyzed in HeLa cells whether the localization pattern of the mutant FMNL2 was changed. Transfection of GFP-tagged variants revealed that wildtype protein displayed a typical localization pattern with rich signal density at the plasma membrane and throughout the length of filopodia, with a distinct signal at the tips of filopodia (as also shown by others e.g. [14,15]). However, the localization pattern of the mutant FMNL2 was clearly different with a strong loss of plasma membrane localization and increased localization within the cell body (Fig 2A).

Using HeLa cells, we also observed that FMNL2 p.L136P expression resulted in an obvious smooth cell surface. Instead, cells expressing the wildtype form displayed a very “hairy” phenotype, indicating active filopodia dynamics in these cells. Under microscopy the difference between these two phenotypes was striking even before quantification analysis. For statistical representation of this impression, we quantified the number and length of individual filopodia in > 10 cells per condition; this analysis revealed that WT FMNL2 expression induces filopodia formation when compared to GFP expression; filopodia numbers and length were strikingly reduced upon expression of the FMNL2 p.L136P mutant (Fig 2B). FMNL2 is known to control filopodia formation in different cell lines [14,30,31]. Filopodia play an important role in sensing of the environment and substrate tethering during different cell functions such as adhesion or migration, in several cell lines they are relevant for the early phase of spreading [32]. In fibroblasts, filopodia controlled by formins are described to probe the surface during substrate occupation [33].

A depletion of the diaphanous autoregulatory domain (DAD) did not lead to noticeable change of the localization pattern. The formin-mutant maintained its apparent localization to the cytosol compartment (Fig 2C).

Our data indicate that the FMNL2 p.L136P mutation affects not only the subcellular localization but also the functional output.

With the background of our initial findings, we further studied the influence of the formin-mutation on the cytoskeleton-dependent processes in functional assays. For this we first used NIH/3T3 cells, a mouse embryonic fibroblast cell line.

**Fig 3:**
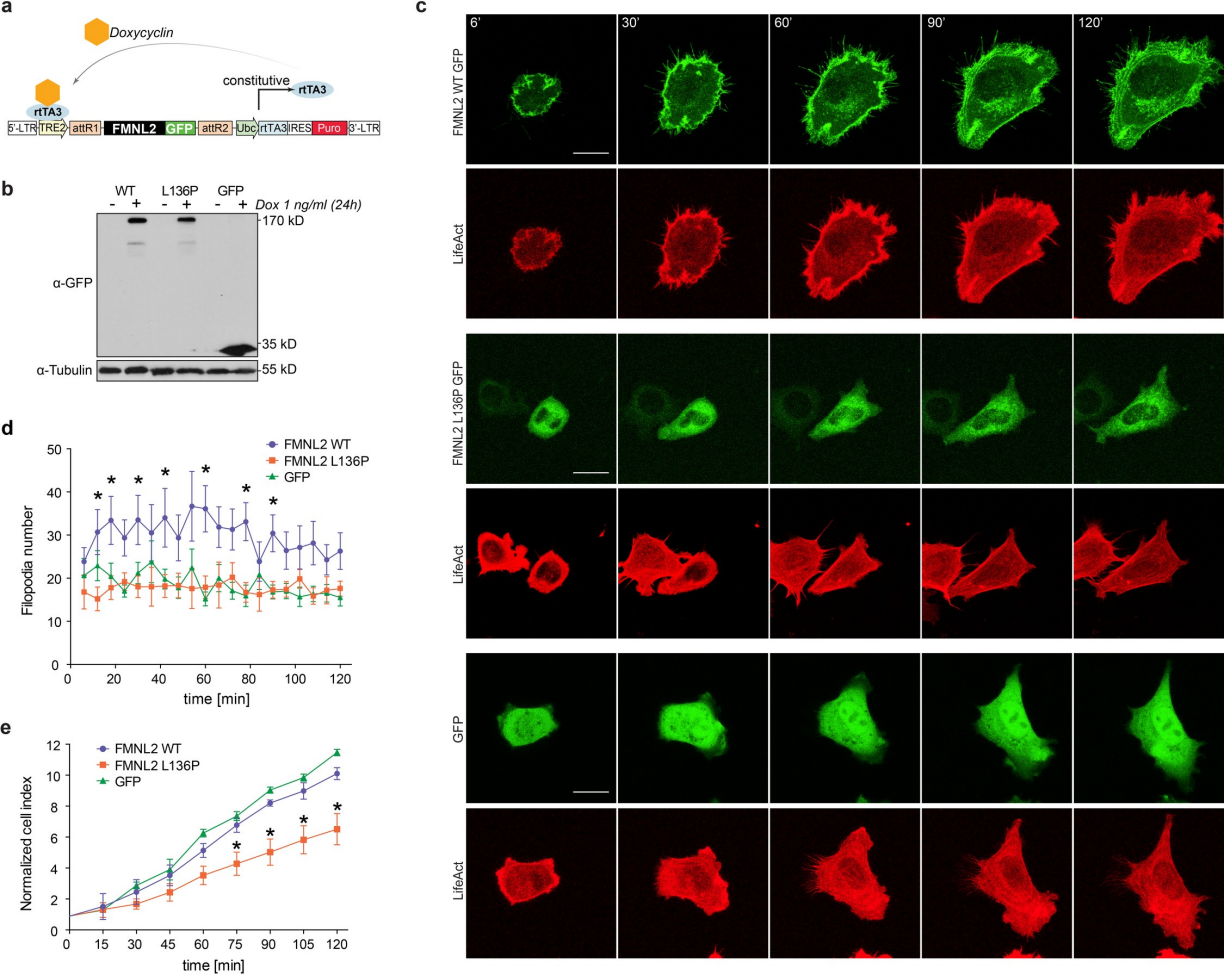
L136P disturbs cell spreading and filopodia formation. **a)** The pInducer20 Puro expression vector [21] allowed for a doxycycline-induced expression of the FMNL2-GFP variants and the appropriate GFP-control. Doxycycline acts as a cofactor for constitutively expressed reverse tetracycline-controlled transactivator (rtTA3) at the tetracycline response element (TRE2). Following lentiviral transduction, puromycin selection was made possible by the expression of a puromycin resistance gene (Puro). (modified from [21]). **b)** The Immunoblot shows the inducible expression of the indicated gene variants in stable NIH/3T3 cell lines without (-) and with (+) Doxycyclin treatment as indicated. **c)** Live cell imaging of NIH/3T3 cells was performed to visualize the dynamics of the spreading process. The cells stably expressed the indicated inducible GFP-tagged variants and LifeAct for visualization of actin-filaments. Stills represent the indicated time points over the course of a 120 minute imaging time. Scale bar = 20 μm **d)** Image-based quantification of filopodia formation during the 120 min spreading process in NIH/3T3 cells (as shown in b). *n = 10*. (p-values for individual time points, L136P vs. WT: 12’ *p = 0*,*017*; 18’ *p = 0*,*022*; 30’ *p = 0*,*023*; 42’ *p = 0*,*044*; 60’ *p = 0*,*007*; 78’ *p = 0*,*004*; 90’ *p = 0*,*012*). **e)** Impedance analysis was performed to assess the dynamic aspects of cell spreading. 5.000 NIH/3T3 cells were seeded in triplicates on 96-well E-plates and impedance measurements were taken every 15 minutes. Normalized cell index indicates the relative increase of impedance that correlates with the mean area of the plated cells (p-values of individual time points, L136P vs. WT: 75’ *p = 0*,*048*; 90’ *p = 0*,*022*; 105’ *p = 0*,*039*; 120’ *p = 0*,*029*).

The generation of stable NIH/3T3 cell lines with transduction of the inducible *pInducer20* vector system [21] allowed for a controlled expression of the genes of interest (Fig 3A and 3B). Through the constitutive coexpression of LifeAct mCherry [34] a parallel visualization of actin filaments was made possible. Cell spreading depends on a controlled dynamic reorganization of the actin cytoskeleton [35]. NIH/3T3 cells expressing the indicated constructs and LifeAct mCherry were subjected to live cell imaging to study the process of cell spreading.

For NIH/3T3 fibroblasts three different spreading phenotypes have been described: ‘filopodial’, ‘smooth-edged’ and ‘ruffled’ [36]. In live cell imaging of the spreading process a ‘filopodial’ driven spreading phenotype could be observed in the wildtype condition. In contrast, p.L136P expressing fibroblasts showed a different spreading phenotype that could be rather characterized as ‘smooth-edged’ or temporarily ‘ruffled’. In addition, p.L136P expressing cells displayed a smaller cell area (S1 Fig). As already observed in HeLa cells, the p.L136P mutant revealed a cytoplasmic localization pattern also in NIH/3T3 cells, whereas the wildtype proteins displayed a clear membrane localization and pronounced filopodia formation (Fig 3C and 3D, S1 and S2 Movies), which supports our previous findings. To assess the dynamic aspects of spreading in an objective manner, impedance assays of NIH/3T3 cells were performed. Sequential measurements every 15 minutes allowed a quantitative analysis of changes in the total cell area, which was correlated to the recorded increase of impedance. To ensure valid quantification the initial impedance values were normalized and data were recorded as ‘normalized cell index’ which indicated the relative increase in total cell area. From 75 minutes, L136P expressing cells showed a significant reduction in normalized cell index at all time points (Fig 3E). These data substantiate the previous findings by demonstrating the negative effect of L136P on different actin-dependent processes.

All Data are shown as mean + SEM of 3 independent experiments. Students T-test was used to analyze the statistical difference between two groups (* = *p<0*,*05*).

Since L136P showed a negative effect on filopodia formation in HeLa cells, we also quantified filopodial protrusions in NIH/3T3 cells during the spreading process. We could observe a significantly reduced number of filopodia at most of the analyzed timepoints in comparison with the wildtype control (Fig 3D). However, GFP expressing controls showed a similar number of protrusions as the L136P condition whereas the spreading area was not affected. These results slightly differ from the observations of filopodia formation in HeLa cells. This could be explained by several factors. Despite the low cell number counted, the significantly altered filopodia number in the mutant cells could be reproduced, however there is a significant discrepancy in the extend of the effect. It is therefore important to point out the differences in the methodic procedures. The HeLa cells were kept on acid-treated glass coverslips and fixed and stained before imaging. The NIH/3T3 cells were transferred onto untreated 8-well μ-slides and imaged during the spreading process. So, the different cell lines and the difference in the surface, e. g. resulting in differences in adherence response, could be an explanation for the diverging results. Also, for the initial studies on subcellular localization, the HeLa cells were transfected with the respective constructs whereas NIH/3T3 cells underwent lentiviral transduction to establish stable cell lines. The expression levels of the transient transfection were significantly higher than in stable NIH/3T3 cell lines with induced expression of the formin variants. Additionally, it has to be mentioned that the formation of cell protrusions in the WT expressing NIH/3T3 cells reaches a peak after around 60 minutes of process spreading, whereas in the late phases between 90 and 120 minutes the counted filopodia number drops. This could be due to an upregulated formation of actin-based protrusions during the active spreading process, facilitated by the overexpression of WT FMNL2.

These data indicate that the initial formation of filopodial structures might not be affected. However, the filopodia that are formed in the context of the FMNL2 mutant seem to be less productive; as cell spreading, as a consequence of filopodia formation, was significantly reduced by the expression of FMNL2 L136P.

We suspect that uncontrolled protein-activity within inappropriate compartments of the cell could disturb the control of physiological actin-dynamics. Probably this causes a deficient spreading dynamic as well as a different phenotype with a reduced formation of actin-based protrusions.

### FMNL2 L136P affects podosome formation and matrix degradation in THP-1 macrophages

As the mutation was found in the peripheral blood of a patient, we assessed the effect of the FMNL2 mutant in a more relevant cell culture model. To this end we used THP-1 cells, a human monocyte cell line that is derived from acute monocytic leukemia. THP-1 cells can adopt a macrophage-like state upon treatment with PMA [22], which is morphologically characterized by an adherent state and the formation of podosomes. Podosomes are actin-dense structures that can mediate the digestion of extracellular matrix (ECM) by matrix-metalloproteinases [37]. Degradation of the ECM is a relevant functional component in transmigration of macrophages [38].

**Fig 4:**
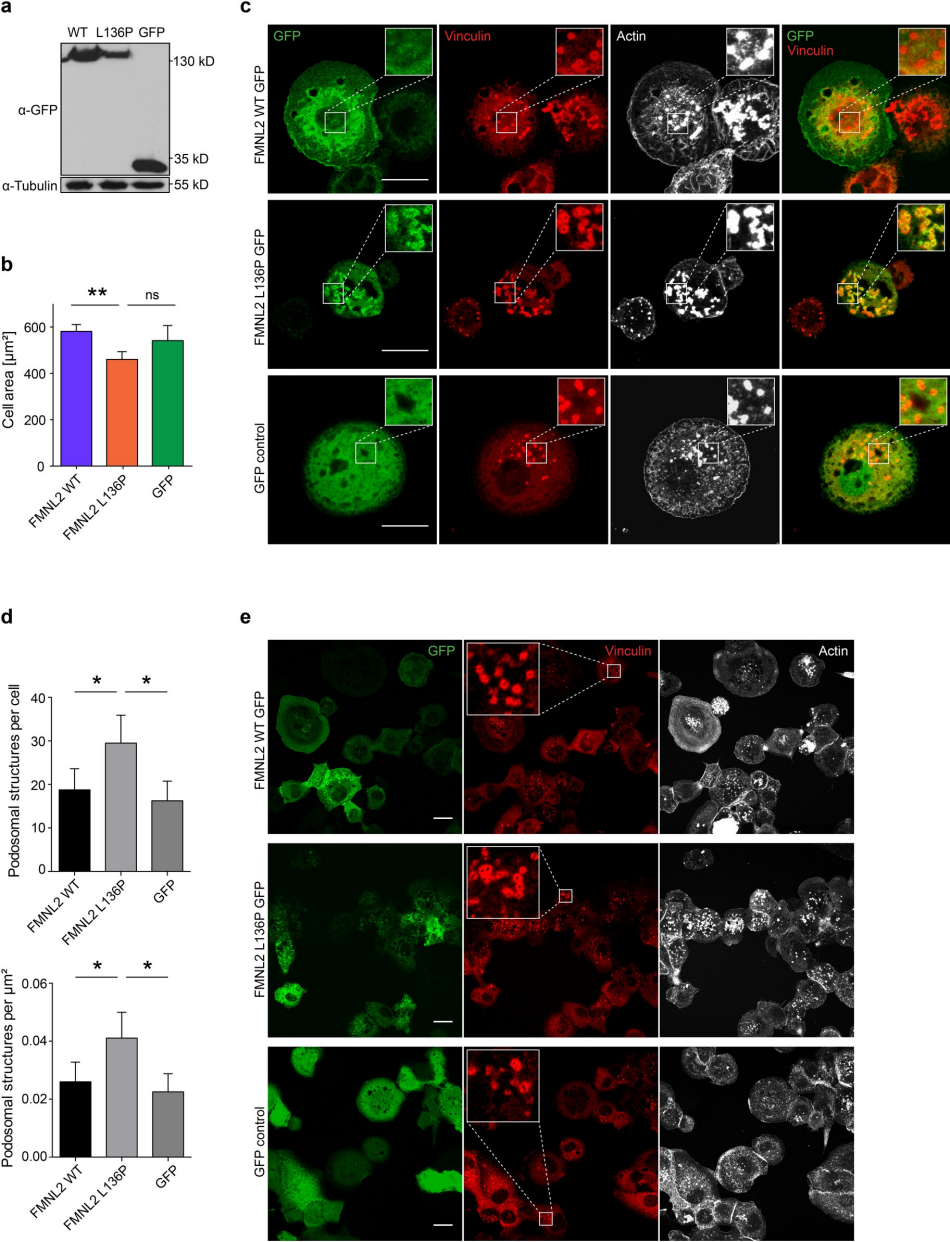
FMNL2 L136P affects podosome-like structures in THP-1 macrophages. **a)** Stable THP-1 cell lines expressing the indicated FMNL2 GFP constructs were generated using lentiviral transduction. **b)** Spreading of differentiated THP-1 cells after 60 minutes. Cells were treated with PMA, diluted by scraping and subsequently seeded on untreated glass coverslips. After 60 minutes cells were fixed and stained with Phalloidin. Random tile scan images were taken and the cell area was measured in > 50 cells per experiment. (L136P vs. WT: *p = 0*,*009*; GFP vs. L136P: *p = 0*,*127*). Data are shown as mean (+SEM) of 3 independent experiments. **c)** THP-1 cells were kept on untreated glass coverslips and differentiated with PMA. Fixed cells were stained with Phalloidin to visualize actin structures and anti-vinculin as a marker for podosome-like structures. FMNL2 L136P shows less plasma membrane association and a condensed signal at vinculin-positive podosome-like structures (as demonstrated in the zoomed-in close-up images). Actin foci are characteristic of the core of podosomes [24]. Scale bar = 20 μm. **d)** Image-based quantification of Vinculin-positive podosomal structures per cell (L136P vs. WT: *p = 0*,*037*; L136P vs. GFP: *p = 0*,*015*) and per μm^2^ cell area (L136P vs. WT: *p = 0*,*036*; L136P vs. GFP: *p = 0*,*014*). Cells were kept on glass coverslips and treated with PMA. Following fixation and staining with vinculin-antibody and phalloidin, for each condition random tile scan images were taken and vinculin-positive ring-formed structures were counted as podosomal structures in > 58 cells. Data are shown as mean (+SD) of 4 independent experiments; at least 58 cells per condition were analyzed. **e)** Representative images showing the differences in podosome number and density as presented in d). Close-up images show vinculin-positive ring structures that are characteristic of podosomes [24]. Scale bar = 20 μm.

THP-1 were stably transduced with constitutive expression vectors for FMNL2 (Fig 4A). Expression of FMNL2 L136P resulted in a smaller spread area (Fig 4B) as also seen for NIH/3T3 cells. Interestingly, in image analysis we could observe that the mutant FMNL2 displayed a condensed signal at podosomal structures, whereas WT FMNL2 and the GFP-control showed no enhanced signal at vinculin-positive foci (Fig 4C). Also, the expression of FMNL2 L136P resulted in an increased number and density of vinculin-positive actin foci per cell (Fig 4D and 4E). Podosomes are punctual structures that mediate matrix digestion in macrophages and share many structural similarities with invadopodia [39]. At the core of podosomes dynamic actin turnover takes place [40]. Known players in controlling podosome associated actin-dynamics are Cdc42, VASP and the Arp2/3-complex [38]. In the formin family FMNL1, INF2 and FHOD1 could be related to podosomes [41,42]. So far, FMNL2 could only be described in the context of actin polymerization at invadopodia, controlled by Cdc42 [43]. In these experiments we used vinculin as a marker for podosomal structures. Vinculin is prominently present at the characteristic ring of podosomal structures [24]. However, it has to be considered that this does not serve as a sufficient marker to evaluate the functionality of podosomes.

**Fig 5:**
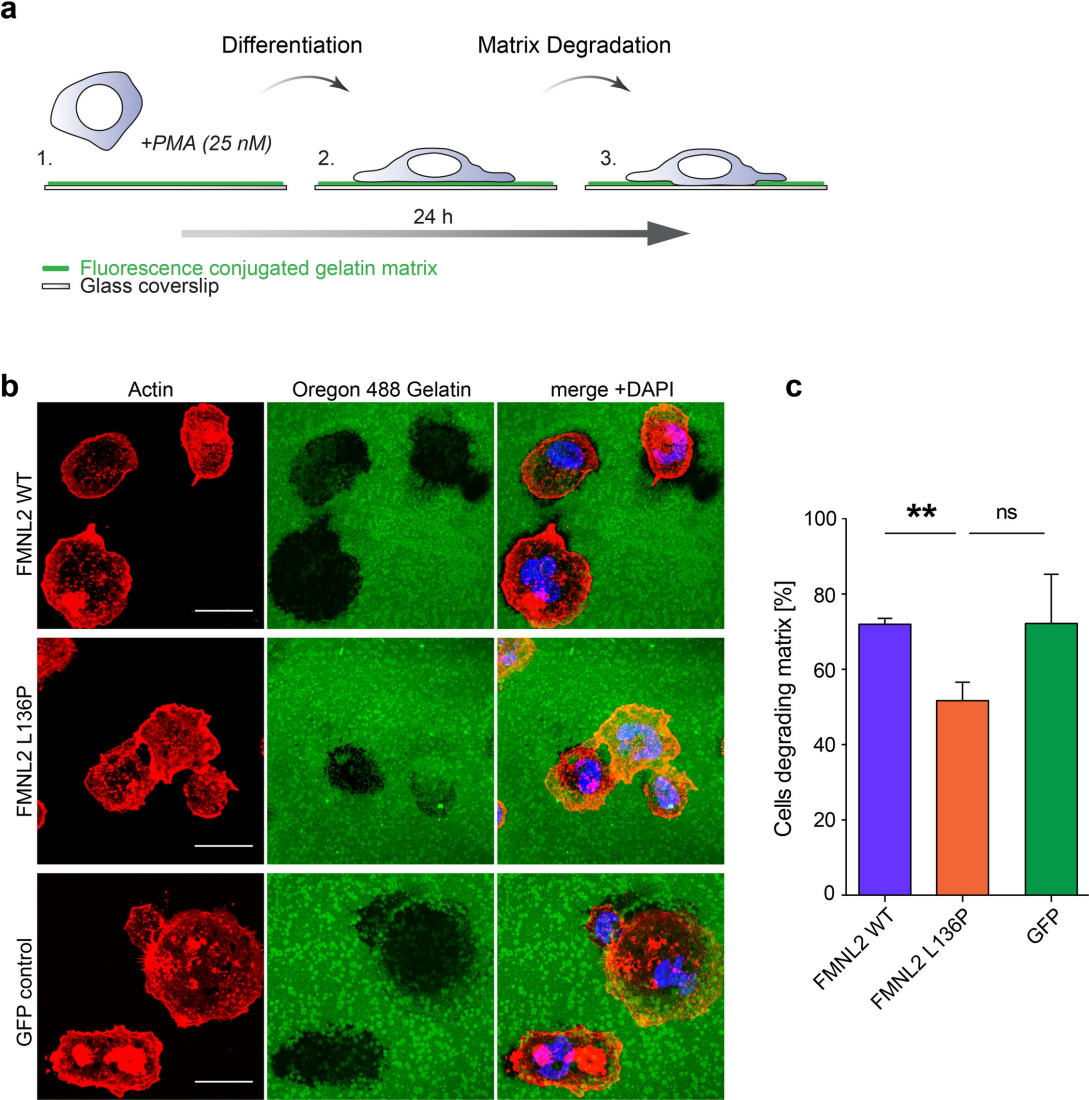
The formin mutant disturbs matrix degradation in THP-1 macrophages. **a)** Schematic cartoon of the matrix degradation assay. THP-1 cells were seeded on fluorescence labeled gelatin matrix with PMA treatment over 24 hours. PMA stimulates the differentiation from monocyte into macrophage state. Dynamic podosome activity enables macrophages to degrade the matrix over time via enzymatic digestion. **b)** Matrix degradation of THP-1 macrophages. THP-1 cells were seeded on glass coverslips coated with fluorescence-labeled gelatin matrix. After fixation cells were stained with Alexa Phalloidin to visualize actin structures. Digested matrix appears black in the green gelatin matrix layer. Scale bar = 20 μm. **c)** Quantification of cells showing degradation activity (black areas in the fluorescent matrix) was performed on tile scan images representing > 100 cells per experiment. Data are shown as mean (+SEM) of 3 independent experiments (L136P vs. WT: *p = 0*,*002*; L136P vs. GFP: *p = 0*,*064*). Students T-test was used to analyze the statistical difference between two groups (* = *p<0*,*05*; ** = *p<0*,*01*).

To assess podosome function, gelatin matrix degradation assays were performed. THP-1 cells were seeded on fluorescent gelatin-coated cover slips and differentiated for 24 hours (Fig 5A). After fixation and staining, we quantified the fraction of cells showing degrading activity (Fig 5B). Strikingly, macrophages expressing FMNL2 L136P showed a significantly decreased fraction of degrading cells. The mean percentage of degrading cells was 72% for cells expressing FMNL2 WT and the GFP control condition, whereas only 52% of FMNL2 L136P expressing cells showed signs of matrix degradation (Fig 5C). This reveals a surprising negative influence of the dysregulated FMNL2 mutant on a relevant macrophage function.

These results indicate a negative effect of the dysregulated FMNL2 L136P mutant on the matrix degradation in THP-1 cells. This is probably due to a disturbed control of actin-dynamics causing uncontrolled actin-polymerization in wrong areas within the cell. However, whether FMNL2 is relevant in podosome-mediated matrix degradation remains unclear and has to be addressed in further investigations. The degradation of ECM serves several complex macrophage functions such as transmigration of tissues or extravasation [38]. As the physiology of monocytes and macrophages is crucial for gut homeostasis [44] these findings point towards a possible contribution of FMNL2 L136P to the immune dysregulation in this case.

From our data it remains unknown why the mutant formin leads to an increased number of podosomal structures although matrix-degradation is significantly compromised. Despite their increased number they seem to lose their functionality. A possible explanation for this could be a disturbed turnover of podosomes caused by dysregulated actin-dynamics, which certainly has to be assessed in further studies.

The patient from which the mutation was identified, suffered from an early-onset disease within his first year of life and a severe disease phenotype throughout the course of his childhood. As early-onset diseases with refractory courses are often related to an immune defect, a primary immunodeficiency was suspected by the treating physicians. Overall, the untypical course suggested a possible influence of the newly identified FMNL2 L136P mutation on the clinical phenotype. However, how the FMNL2 mutant could be specifically involved in the pathogenesis of an immunoregulatory dysfunction remains to be addressed in further studies.

## Supporting information

S1 Fig FMNL2 L136P expressing NIH/3T3 Fibroblasts show reduced cell area after 90 minutes of spreading. a) Confocal imaging of NIH/3T3 cells expressing the indicated constructs after 90 minutes of cell spreading. *Scale bar = 20 μm*. b) Image-based quantification of cell mean areas and the percentage of *“spread cells”* with a cut-off of *cell area > 400 μm2*. Data are show the mean (+SEM) of 3 independent experiments. Students T-test was used to analyze the statistical difference between two groups (* = p<0,05; ** = p<0,01; *** = p<0,001).
(TIF)

S1 Movie Live cell spreading of NIH/3T3 fibroblasts expressing FMNL2 WT. Cells with induced expression of FMNL2 WT GFP were transferred to an incubation chamber. After visual adherence to the bottom of the dish, images were taken every 6 minutes over a period of 108 minutes. Constitutive Co-expression of LifeAct mCherry allowed visualization of actin-filaments. Through the overexpression of the wildtype formin, a filopodia-driven spreading phenotype could be observed.
(AVI)

S2 Movie Live cell spreading of NIH/3T3 fibroblasts expressing FMNL2 L136P. Cells with induced expression of FMNL2 L136P GFP and constitutive co-expression of LifeAct mCherry were transferred to an incubation chamber and were imaged for 108 minutes at a frequency of 6 minutes, according to the wildtype condition. The mutant formin revealed a rather ‘smooth-edged’ spreading phenotype.
(AVI)

S1 Raw images (PDF)
