# Supplementary figures and images for: Characterization of a L136P mutation in Formin-like 2 (FMNL2) from a patient with chronic inflammatory bowel disease
Source: PLoS One. 2021 May 27;16(5):e0252428. doi: 10.1371/journal.pone.0252428 (PMC8158924; doi:10.1371/journal.pone.0252428)

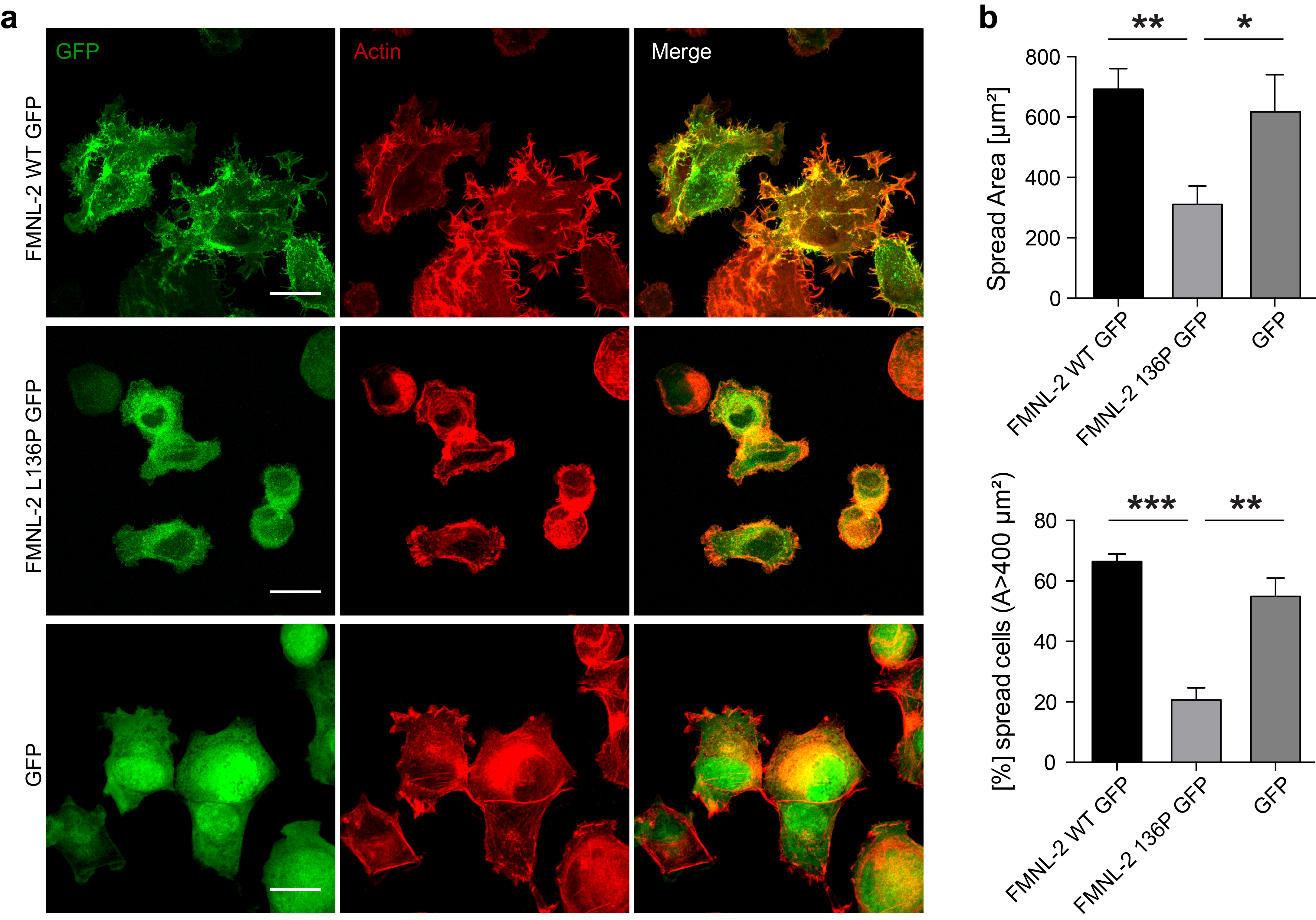

Supplement: S1 Fig — a) Confocal imaging of NIH/3T3 cells expressing the indicated constructs after 90 minutes of cell spreading. Scale bar = 20 μm. b) Image-based quantification of cell mean areas and the percentage of “spread cells” with a cut-off of cell area > 400 μm2. Data are show the mean (+SEM) of 3 independent experiments. Students T-test was used to analyze the statistical difference between two groups (* = p<0,05; ** = p<0,01; *** = p<0,001). (TIF) [file pone.0252428.s001.tif]

The HRP-Signal was detected on Medical X-Ray Films (Fuji) using X-Ray Film Processor (Medical Index)

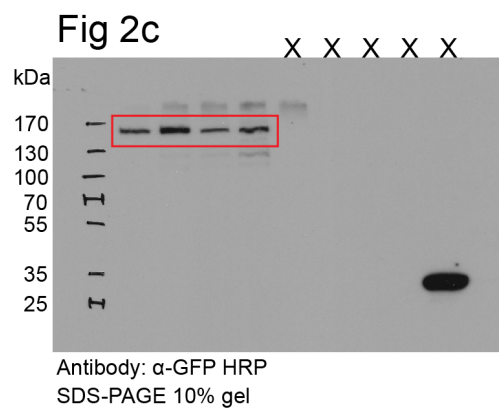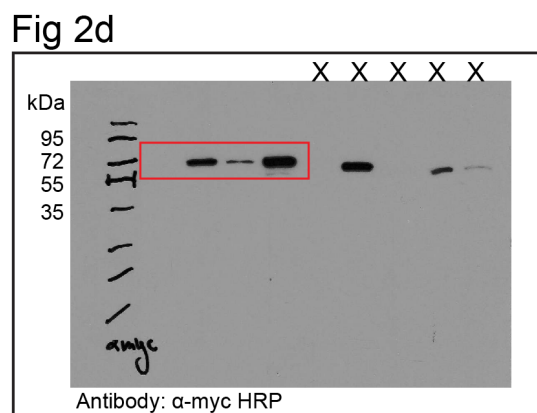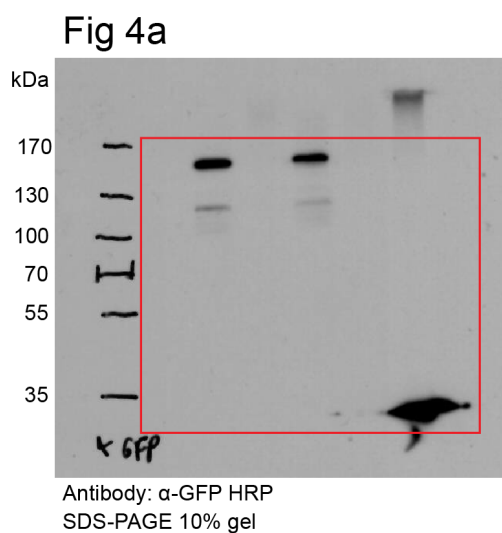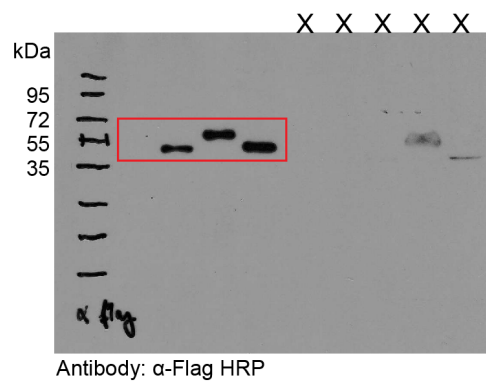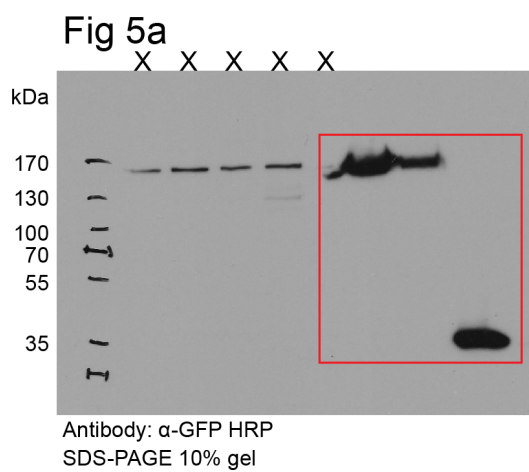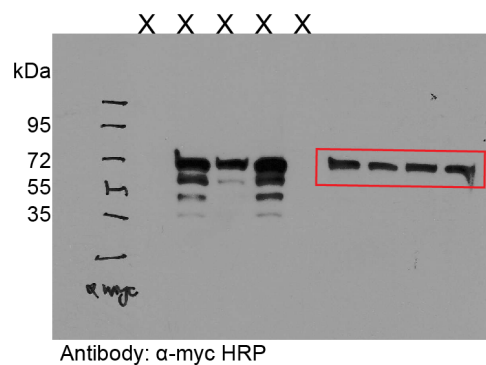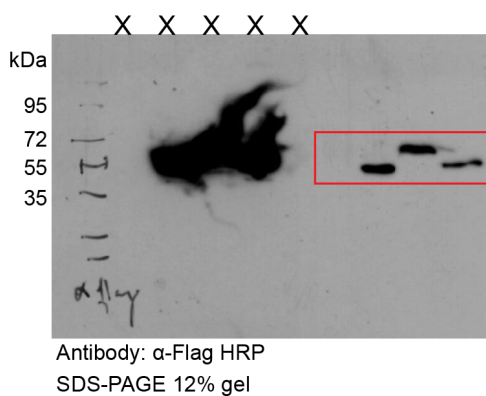

Supplement: S1 Raw images — (PDF) [file pone.0252428.s004.pdf]
